# The treatment of high tibial osteotomy non-union with the Ilizarov external fixator

**DOI:** 10.1007/s11751-012-0138-3

**Published:** 2012-07-01

**Authors:** John J. Gillooly, Konstantinos Tilkeridis, Robert B. Simonis, Fergal Monsell

**Affiliations:** 1 Department of Orthopaedic Surgery, Bristol Royal Infirmary, Upper Maudlin Street, Bristol, UK; 2 Department of Orthopaedic Surgery, Bristol Children’s Hospital, Upper Maudlin Street, Bristol, UK; 3 St. Peter’s Hospital, Guildford Road, Chertsey, Surrey KT16 0PZ UK

**Keywords:** Non-union, High tibial osteotomy, Ilizarov, External fixator, Circular frame

## Abstract

To evaluate the results of the Ilizarov external fixator in the treatment of non-union post–high tibial osteotomy (HTO). Five non-unions, in four patients, following HTO were treated by Ilizarov fixation. Clinical outcome was assessed pre- and post-operatively by the Knee Society Clinical Rating System (KSCRS). Radiological analysis assessed bone healing pre- and post-operatively and measured proximal tibial alignment. All cases healed with a mean time of 25 ± 3 weeks (Mean ± SD) (range, 24–30 weeks) in the fixator. The clinical and radiological outcome improved in all cases. Four knees were initially in excessive varus and underwent correction of alignment, as measured by medial proximal tibial angle (MPTA), from 75.5° ± 8.4° (mean ± SD) to 90.2° ±  2.7° (normal range, 85°–90°). One patient was in excessive valgus and had a correction of MPTA from 100° to 87°. The KSCRS knee score improved from 35.6 ± 10.8 to 86.6 ± 13.9 (mean ± SD) (normal score = 100) and the functional score from 37.8 ± 11.8 to 85.4 ± 10.5 (mean ± SD) (normal score = 100). The Ilizarov technique is a minimally invasive method that produces excellent clinical, radiological and functional outcomes.

## Introduction

The high tibial osteotomy (HTO) remains an established procedure for patients with painful medial compartment osteoarthritis who are considered too young for knee arthroplasty. It is also used, although less commonly, in older patients who are reluctant to undergo an arthroplasty [1–3]. Closing-wedge, and dome osteotomies are commonly used techniques, but alternative techniques including the medial opening-wedge osteotomy [4] or high tibial osteotomy using a circular external fixator [5] have been described.

A non-union is a rare complication of the HTO. Previous studies describing treatment methods with conventional fixation have reported mixed results. This study describes the use of the Ilizarov external fixator (IEF) on patients who were unsuitable for other forms of operative intervention due to the small size of the proximal tibial fragment.

## Materials and methods

The cohort comprised patients with failed open operative intervention on the non-union site or those where open intervention was not considered to be technically feasible due to the small size of the proximal fragment. Five non-unions in four patients were treated with the IEF from 1996 to 2005. The mean patient age was 51 years (range, 18–65). One patient had bilateral tibial osteotomies and both failed to unite. Three patients (four non-unions) had retained hardware from the initial HTO, which was removed prior to the application of the fixator. In four knees, there was a varus deformity and in one, a valgus deformity. None of the non-unions were infected.

There were four hypertrophic (stiff) non-unions and one atrophic (partially mobile) non-union. The patients’ demographic details and period of treatment are summarized in Table 1. The initial osteotomy type, method of fixation, subsequent surgical attempts at achieving union prior to use of the IEF and details of adverse clinical incidents are recorded in Table 2. Clinical outcome was assessed with the Knee Society Clinical Rating System (KSCRS) [6]. Radiographic analysis consisted of assessment of alignment using the methods described by Paley and Tetsworth [7] and the Insall–Salvati ratio [8].

**Table 1:** Demographic data of the patients

Case	Non-union type	Deformity	Sex	Age	Smoking	Time in frame (weeks)
1	Hypertrophic—stiff	Varus	M	18	No	30
2	Hypertrophic—stiff	Varus	M	57	Yes	20
3	Atrophic—part mobile	Valgus	F	64	Yes	24
4	Hypertrophic—stiff	Varus	F	65	Yes	26
5	Hypertrophic—stiff	Varus	F	65	Yes	24

**Table 2:** Initial high tibial osteotomy, method of fixation, revision procedures prior to the application of the Ilizarov external fixator (IEF) and complications of the IEF

Case	Initial osteotomy	Implant used	Subsequent surgery	Complications of the IEF
1	Dome	Plaster immobilization	None	Sinus requiring debridement
2	Opening wedge	Puddu plate (Broke 6 months post-op)	Dome osteotomy with Puddu plate	Superficial pin site infection
3	Dome	Staples	None	Superficial pin site infection
4	Opening wedge	AO plate	None	Superficial pin site infection
5	Opening wedge	AO plate	None	Replacement of broken wire

**Fig. 1:**
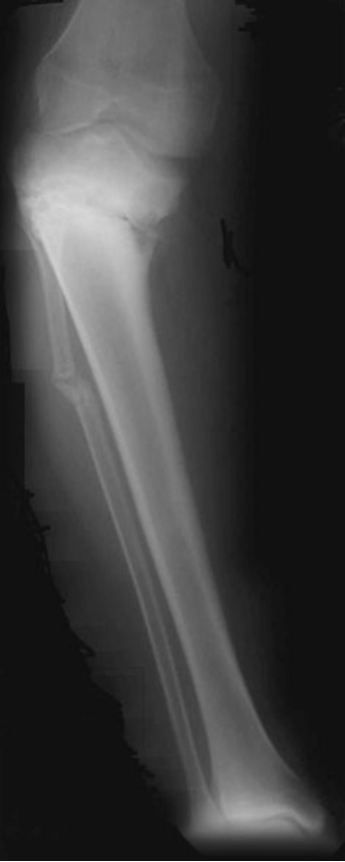
Pre-operative high tibial osteotomy non-union; varus and 2 cm short

**Fig. 2:**
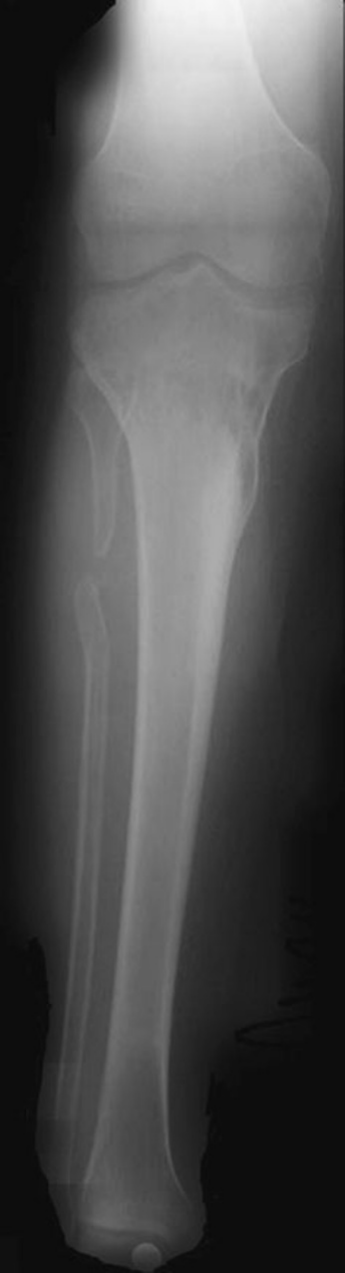
Post-Ilizarov alignment with a 2-cm leg length correction

The lEF (Smith and Nephew Orthopaedics, Memphis, TN) was used in all cases with multiple opposed 1.8-mm olive wires inserted under fluoroscopic guidance. The proximal fragment was short in all cases, and one ring was used for this segment. Two rings were attached to the longer distal tibial fragment. A fibular osteotomy was performed in all cases to facilitate angular correction. The four cases of stiff non-union underwent gradual angular correction to neutral [9] and followed by a period of compression at the non-union site until bone healing was demonstrated radiologically. The atrophic non-union was sufficiently mobile to acutely correct the varus angular deformity so that the non-union could be compressed immediately. In one case, there was 2 cm shortening after the varus deformity had been corrected by the IEF. This was eliminated by the distraction through the non-union, and healing was established after 6 months with equal leg lengths (Figs. 1, 2).

## Results

All non-unions healed. The mean follow-up was 66 months (range, 6–144). The patients were uniformly satisfied with the results of the procedure and, when asked, elected to receive the same treatment again if necessary. All patients had an improvement in Knee Scores: The mean pre-operative KSCRS score was 35.6 ± 10.8 (±SD) and this improved to a mean post-operative score of 86.6 ± 13.9 (±SD). The mean pre-operative functional score was 37.8 ± 11.8 (±SD) and improved to a mean score of 85.4 ± 10.5 post-operatively (Table 3). The mean time in the IEF was 25 ± 4 weeks (±SD, range, 24–30 weeks). All patients maintained full knee extension, and there was an improvement in knee flexion by an average of 10°. One patient underwent 2 cm limb lengthening for a leg length discrepancy (Table 4).

**Table 3:** Pre- and post-operative knee society score [6]

Case	Knee score		Functional knee score	
	Pre-op	Post-op	Pre-op	Post-op
1	24	100	20	86
2	42	63	33	69
3	24	90	48	84
4	41	90	40	97
5	47	90	48	91

**Table 4:** Pre- and post-operative knee angles and leg length

Case	MPTA^a^		PPTA^b^		Length (cm)	
	Pre-op	Post-op	Pre-op	Post-op	Discrepancy	Lengthening
1	63	89	64	75	2	2
2	81	87	94	86	0	0
3	100	87	87	87	0	0
4	80	92	91	90	0	0
5	78	93	92	87	0	0

^a^Medial proximal tibial angle [7]

^b^Posterior proximal tibial angle [7]

The final alignment was satisfactory in all cases. In four patients where the non-union was in varus, the mean pre-operative MPTA was 75.5° ± 8.4° (mean ± SD) (normal range, 85°–90°). This improved to a mean post-operative MPTA of 90.2° ± 2.7°. One patient in valgus pre-operatively had a correction of MPTA from 100° to 87° (Table 4). The mean pre-operative posterior proximal tibial angle (PPTA) was 85.6° ± 12.3 (mean ± SD) (normal range, 77°–84°) and this remained essentially unchanged at 85.4° ± 10.5 (mean ± SD) at the end of treatment (Table 4). The mean Insall–Salvati ratio pre- and post-operatively was 1.01 and 1.04, respectively, thus remaining within normal limits.

Four patients developed superficial pin site infections during treatment, which responded to oral antibiotics. One patient developed a sinus at a pin site following removal of the frame, which was successfully treated by a surgical debridement and a 3-day course of intravenous flucloxacillin. Repeat X-ray and inflammatory markers at 6 months demonstrated no evidence of continued infection or bony involvement. Replacement of a single wire, which failed three months after IEF application, was required in one patient.

## Discussion

Non-union after high tibial osteotomy is an unusual complication [10–12] as the proximal tibial metaphysis is a broad surface of well-vascularized cancellous bone with good healing potential. Coventry reported a series of 213 osteotomies with no non-unions [11]. Hsu reported only one non-union in a series of 118 Maquet dome osteotomies secured with Charnley’s external compression fixators, and Bauer et al. reported one non-union in 66 cases [10, 12]. The metaphyseal location of the non-union after high tibial osteotomy can make stable fixation problematic because the proximal fragment is small and difficult to control with most conventional methods of internal fixation. Resection of the pseudarthrosis, external fixation with Charnley’s compression clamps followed by casting, was proposed by Tjornstand et al. in one of the first series of HTO non-unions [13]. Although this resulted in bony union, resection of the pseudarthrosis in one of their patients resulted in such a thin proximal tibial fragment that compression had to be applied across the knee joint.

Single plating of the non-union has been described but produced poor results, and although superior rates of union were achieved in the same study with a double plating technique, this produced only fair success rates [14]. Wolff and Krackow reported the results of internal fixation in a series of six patients with non-union after HTO [15]. They stressed the importance of the preservation of metaphyseal bone stock for future TKR and routinely performed open bone grafting. Schatzker et al. reported three cases successfully managed with bone grafting and compression with an AO external fixator [16]. Rozbruch et al. used the principles of distraction osteogenesis in five hypertrophic non-unions after HTO, and they highlighted the benefits of using a minimally invasive technique to achieve a good clinical and radiological outcome [17]. Although they included 2 cases treated by an IEF, these were mixed with 3 patients treated with a larger 6-mm monolateral fixator, thereby preventing analysis of the IEF outcomes.

Our series specifically reports the results of the IEF in the treatment of this uncommon complication. We have demonstrated that this technique was successful in achieving bony union in five non-unions, which were not amenable to internal fixation due to the size of the proximal fragment and magnitude of deformity, through a percutaneous approach and with minimal blood loss. As well as facilitating bony healing, the IEF also allowed simultaneous correction of the deformity. In one patient, distraction histogenesis techniques were used to correct the angular and axial deformity. In the case of atrophic non-union, compression was applied whilst maintaining alignment.

As the HTO is often a stop-gap procedure before a knee arthroplasty, there is always concern when managing HTO non-unions as to what effect treatment will have on the outcome of a future arthroplasty. The long-term results of total knee replacement (TKR) in limbs that have had a previous proximal tibial osteotomy have been shown to be slightly inferior [18]. This is possibly related to malalignment, previous skin incisions, patella baja and bone abnormalities, which could significantly reduce the bone available during the TKR. The IEF should have minimal impact on future TKR, as it is a percutaneous application; the bone stock is preserved as the pseudarthrosis is left in situ and the underlying deformity is corrected through angular distraction and a normal Insall–Salvati ratio for the patella position maintained. There is a theoretical concern that the thin proximal tibial fragment may lead to the intra-articular placement of wires, leading to an increased risk of septic arthritis and osteomyelitis [19]. This did not occur in any of the patients in this study. However, single episodes of superficial pin site infections were seen in four of the five knees in our series. All were treated successfully with 7-day courses of flucloxacillin.

The aims of treatment for patients with non-union following a HTO are bony union, axial alignment, correction of leg length discrepancy and bone stock preservation for a future TKR; all were achieved using the minimally invasive technique with an IEF.
